# Tumor regression mediated by oncogene withdrawal or erlotinib stimulates infiltration of inflammatory immune cells in EGFR mutant lung tumors

**DOI:** 10.1186/s40425-019-0643-8

**Published:** 2019-07-10

**Authors:** Deborah Ayeni, Braden Miller, Alexandra Kuhlmann, Ping-Chih Ho, Camila Robles-Oteiza, Mmaserame Gaefele, Stellar Levy, Fernando J. de Miguel, Curtis Perry, Tianxia Guan, Gerald Krystal, William Lockwood, Daniel Zelterman, Robert Homer, Zongzhi Liu, Susan Kaech, Katerina Politi

**Affiliations:** 1 Department of Pathology, Yale School of Medicine, 333 Cedar Street, SHM-I 234D, New Haven, CT 06510 USA; 2 Yale Cancer Center, Yale School of Medicine, New Haven, CT 06510 USA; 3 Department of Immunobiology, Yale School of Medicine, New Haven, CT 06510 USA; 4 Present address: Department of Fundamental Oncology, University of Lausanne, Ludwig Cancer Research Lausanne Branch, Lausanne, Switzerland; 5 British Columbia Cancer Agency, B.C, Vancouver, V5Z 1L3 Canada; 6 Department of Biostatistics, Yale School of Public Health, New Haven, CT 06510 USA; 7 VA Connecticut Healthcare System, Pathology and Laboratory Medicine Service, 950 Campbell Ave, West Haven, CT 06516 USA; 8 Present address: Salk Institute for Biological Studies, La Jolla, CA 92037 USA; 9 Department of Medicine (Section of Medical Oncology), Yale School of Medicine, New Haven, CT 06510 USA

**Keywords:** Lung cancer, EGFR, Targeted therapies, Immunotherapies, Mouse models

## Abstract

**Background:**

Epidermal Growth Factor Receptor (EGFR) tyrosine kinase inhibitors (TKIs) like erlotinib are effective for treating patients with *EGFR* mutant lung cancer; however, drug resistance inevitably emerges. Approaches to combine immunotherapies and targeted therapies to overcome or delay drug resistance have been hindered by limited knowledge of the effect of erlotinib on tumor-infiltrating immune cells.

**Methods:**

Using mouse models, we studied the immunological profile of mutant EGFR-driven lung tumors before and after erlotinib treatment.

**Results:**

We found that erlotinib triggered the recruitment of inflammatory T cells into the lungs and increased maturation of alveolar macrophages. Interestingly, this phenotype could be recapitulated by tumor regression mediated by deprivation of the EGFR oncogene indicating that tumor regression alone was sufficient for these immunostimulatory effects. We also found that further efforts to boost the function and abundance of inflammatory cells, by combining erlotinib treatment with anti-PD-1 and/or a CD40 agonist, did not improve survival in an EGFR-driven mouse model.

**Conclusions:**

Our findings lay the foundation for understanding the effects of TKIs on the tumor microenvironment and highlight the importance of investigating targeted and immuno-therapy combination strategies to treat *EGFR* mutant lung cancer.

**Electronic supplementary material:**

The online version of this article (10.1186/s40425-019-0643-8) contains supplementary material, which is available to authorized users.

## Background

*EGFR* mutations are found in 10–15% of lung adenocarcinomas in the US and are enriched in tumors from never or former smokers [1]. Lung adenocarcinoma-associated mutations in exons encoding the tyrosine kinase domain of this receptor most commonly include either deletion of a four amino acid motif (LREA) in Exon 19 of *EGFR* or a point mutation in Exon 21, which substitutes Arginine for Leucine at position 858 (L858R) [2]. These mutations confer sensitivity to EGFR tyrosine kinase inhibitors (TKIs) such as erlotinib, gefitinib and afatinib, current standard of care therapies for the treatment of this subset of lung cancer. However, drug resistance inevitably develops on average after 12 months of treatment [3, 4]. In more than 50% of cases, acquired resistance to erlotinib is driven by a second site mutation in EGFR, T790M [3, 5], which alters the affinity of the receptor for ATP and as a consequence to the drugs [6]. Novel 3rd generation TKIs that specifically inhibit mutant EGFR (and spare wild-type EGFR) are now also approved to treat this disease in both the first and second line settings to overcome and/or delay the onset of resistance [7]. Even with these improvements, however, none of the therapies are curative [8]. Therefore, demands for novel therapeutic approaches are high.

Recent advances show that targeting the immune system is a useful approach to treating lung cancer. Mounting evidence suggests that tumors stimulate the establishment of an immunosuppressive microenvironment to evade the immune system by facilitating tumor-infiltrating T cells to display an exhausted phenotype [9] such that they are unable to proliferate and produce pro-inflammatory cytokines [10, 11]. Agents that target inhibitory molecules (e.g. PD-1, CTLA4) on T cells and/or their cognate ligands (e.g. PD-L1) on tumor and immune infiltrating cells have shown promising results in treating lung cancers and are now FDA-approved. However, overall there appears to be a lower response rate to PD-1 axis inhibitors associated with *EGFR* mutations. In a retrospective evaluation of patients treated with PD-1 or PD-L1 inhibitors, it was found that objective responses in patients with *EGFR*-mutant tumors was 3.6% compared to 23.3% in those with *EGFR* wild-type tumors [12]. In spite of this, there are clear indications that a subset of patients with *EGFR* mutant lung cancer benefit from these therapies [13–15]. Moreover, preclinical models demonstrate that the immune system plays an important role in modulating the growth of *EGFR* mutant tumors [16]. In one study evaluating the combination of erlotinib plus nivolumab, durable tumor regression in both treatment (TKI or chemotherapy) naïve and TKI-treated patients was reported [17] and there are several additional trials evaluating the efficacy of combining PD-1/PD-L1 inhibitors with EGFR TKIs [13]. However, toxicities have raised concerns that treating patients with EGFR TKIs and immune checkpoint inhibitors concurrently may not be the optimal approach to use these agents in combination. Given these findings, studies are necessary to understand the effects of EGFR TKIs on the tumor microenvironment and the immunological consequences of combining immune checkpoint inhibitors with EGFR TKIs.

Several studies have examined the effect of kinase inhibitors on the tumor immune microenvironment. The BRAF inhibitor vemurafenib, for instance, has been reported to increase intratumoral CD8^+^ T cell infiltrates [18], increase tumor associated antigens and improve effector function of cytotoxic T lymphocytes [19]. However, a subset of tumors resistant to vemurafenib exhibit features of T-cell exhaustion and reduced antigen presentation suggesting that these may be resistant to checkpoint inhibitors [20]. Similarly, in lung cancer cell lines, two studies have revealed that TKI treatment leads to down-regulation of tumor PD-L1 expression [21, 22]. Moreover, it has also been shown that erlotinib can impair T cell-mediated immune responses via suppression of signaling pathways downstream of EGFR critical for cell survival and proliferation [23]. Further supporting that erlotinib could have immunosuppressive effects on the immune system, erlotinib has been posited to down-regulate TNF-α mediated inflammation characteristic of psoriasis [24]. In addition, a study in mouse models of *EGFR* mutant lung cancer reported increased leukocyte infiltration and enhanced antigen-presenting capabilities after 24 h of erlotinib treatment [25]. While these studies point to modulation of the immune system by TKIs like erlotinib several unanswered questions remain: 1) in addition to the abundance, how is the functionality of the immune cells affected by erlotinib, and specifically of lung-resident immune cells that have not been examined in prior studies? 2) does the immune microenvironment return to normal after tumor regression or are there lingering consequences of the presence of the tumor? 3) are the effects of erlotinib treatment in vivo on the immune microenvironment mediated by erlotinib or are they due to the process of tumor regression? and 4) what are the more long-term effects of erlotinib on the immune microenvironment beyond the effects observed acutely after treatment? To address these issues, we utilized a previously developed immunocompetent mouse model of *EGFR* mutant lung cancer [26] and tested the consequences of erlotinib or oncogene de-induction on the immune microenvironment.

## Methods

### Transgenic mice

*CCSP-rtTA; TetO-EGFR*^*L858R*^ mice were previously described [26]. Mice were fed chow containing doxycycline (625 ppm) obtained from Harlan-Tekland. The animals were housed in a pathogen-free facility and animal studies were performed in accordance with and with the approval of the Yale University Institutional Animal Care and Use Committee (IACUC protocol numbers: 2016–11364, 2016-10806 and assurance number: D16–00416).

### In vivo treatment with Erlotinib

Erlotinib was purchased and purified at the organic synthesis core facility at Memorial Sloan Kettering Cancer Center (MSKCC), dissolved in 0.5% methylcellulose and administered intraperitoneally at 25 mg/kg, 5 days a week. Mice were euthanized by CO_2_ asphyxiation.

### Magnetic resonance imaging

Magnetic resonance images of isofluroane-anesthetized mice were collected using a mini-4 T horizontal-bore spectrometer (Bruker AVANCE). Throughout data collection, each animal was anesthetized on a steady flow of isofluroane and oxygen (2–2.5% v/v) and core-body temperature was maintained at 37 ± 1 °C. Imaging parameters were optimized to effectively discriminate between healthy lung and areas with tumor. Tumor burden in each animal was quantified by calculating the volume of visible lung opacities in every image sequence using the BioImage Suite software [27].

### Tumor digestion

Lungs from normal, untreated, tumor-bearing or treated mice were mechanically digested and incubated in HBSS with 0.5 mg/ml collagenase IV and 1μg/ml DNase 1 at 37 degrees for 1 h after which the solution was filtered using a 70 μm cell strainer. The resulting single cell suspension was incubated in ACK lysis buffer for 5 min to lyse red blood cells.

### Flow cytometry and cell sorting

Single cell suspensions of lung tumors or splenocytes were resuspended in FACS buffer (PBS + 1%FBS). Cells were then incubated with anti-Fc receptor antibody (clone 2.4G2) on ice for 15 min followed immediately by staining with respective surface antibodies for 30 min. For intracellular cytokines, T cells were stimulated with PMA/ionomycin (Sigma Aldrich) and Brefeldin A for 5 h at 37 degrees. The cells were stained first with surface antibodies then fixed in Cytofix/Cytoperm buffer (BD Biosciences) followed by staining with antibodies to detect proteins present in intracellular compartments. FoxP3 staining was done in a similar manner. Samples were acquired on an LSRII flow cytometer and analyzed with Flowjo. Cells were sorted on the BD FACS Aria at the Yale Cell Sorter Core facility. Cells were sorted based on the expression of the following markers: CD4 T cells: CD45+/CD3+/CD4+, CD8 T cells: CD45+/CD3+/CD8+, Alveolar macrophages: CD45+/ CD11c+/SiglecF+, Tumor epithelial cells: CD45−/CD11c-Epcam+.

### In vivo labeling of immune cells

Mice were retro-orbitally injected with 3 μg of biotin-conjugated CD45 (clone 30-F11) for 5 min, immediately after which animals were sacrificed. Lung tissue was collected, processed and stained as described above.

### T cell proliferation assay

Splenocytes and single cell suspensions were collected from spleen or lungs of tumor bearing mice. T cells were enriched using a purified antibody cocktail consisting of IA/I-E, B220 and F4/80. Purified cells were loaded with 5 μM CFSE at room temperature for 15 min in the dark. T cells mixed with anti-CD28 were seeded on CD3 coated plates followed by treatment with 10 μM Erlotinib or DMSO for 5 days. Proliferation was determined by CFSE dilution using flow cytometry.

### Histology, immunofluorescence and cell quantification

Lung tissue from normal, tumor bearing untreated and treated animals was collected after sacrifice, fixed overnight in 4% paraformaldehyde and rehydrated in 70% ethanol until submission for paraffin embedding and sectioning at the Yale Pathology Tissue Services. Sections were stained with hematoxylin and eosin, CD3 (Spring Biosciences; 1:150), EGFR^L858R^ (Cell Signaling; 1:400), FoxP3 APC-conjugated (eBioscience; 1:50), Ki-67 (BioLegend; 1:50) and Cytokeratin 7 (Abcam; 1:300) antibodies. Positive cells in a 40X field of view were manually counted using a plugin for ImageJ called Cell Counter. At least three representative tissue locations were used to quantify and values were averaged for each mouse.

### Bio-Plex Cytokine assay

Healthy lungs or tumors were crushed and homogenized in cold PBS with 1X protease inhibitor cocktail and 1% Triton X-100 (Thermo Scientific). Equal amounts of total protein were analyzed in triplicates using the Bio-Rad Mouse 23-plex cytokine assay (Bio-rad, CA, USA) according to the manufacturer’s protocol.

### RNA extraction, purification and quantitative real-time RT-PCR

For RNA extraction and purification, the Arcturus PicoPure RNA isolation kit was used according to manufacturer’s instruction and cDNA was synthesized using the SuperScript II Reverse Transcriptase from Invitrogen. Quantitative real-time PCR was performed using the Taqman assay (Invitrogen). C_t_ values were recorded and relative gene expression was determined using the ΔΔC_t_ method.

### RNA sequencing and gene expression data

RNA sequencing was performed using the illumina HiSeq 2000 platform through the Yale Stem Cell Center Genomics Core facility. R1 reads from each paired-end reads were aligned to the mouse genome (version mm10) using bowtie2 [28] in local mode, followed by annotation of counts to each gene by gencode (version M10) [29]. Differential expression in each cell type between experimental conditions was performed with the DESeq2 [30] R package.

### Ingenuity pathway analysis

Enrichment analyses of canonical pathways were performed with Ingenuity Pathway Analysis (IPA, Ingenuity Systems). Genes with an adjusted *P* value lower than 0.05 were included and Ingenuity Knowledge Base (Genes Only) was used as reference set for the analyses.

### Statistical analysis

Statistical analysis was performed using GraphPad Prism 7.0 software and *p*-values, where indicated, were determined using the parametric, student’s t-test.

### In vivo treatment with erlotinib, agonistic anti-CD40 antibody and anti-PD-1 antibody

Tumor bearing EGFR^L858R^ mice were treated with erlotinib alone or in combination with an agonistic anti-CD40 antibody and/or anti-PD-1 antibody. Erlotinib (obtained from the Organic Synthesis Core Facility at Memorial Sloan Kettering Cancer Center) was suspended in 0.5% (w/v) methylcellulose. The agonistic anti-CD40 antibody and anti-PD-1 antibody (both from BioXcell) were diluted in PBS. Erlotinib was administered intraperitoneally at 25 mg/kg per mouse, 5 days a week while the agonistic anti-CD40 antibody and anti-PD-1 antibody were administered intraperitoneally at 250 μg/mouse, every 3 days. Tumor volume was assessed by MRI before, during and after treatment duration and at the end of study, mice were euthanized by CO_2_ asphyxiation.

## Results

### Increased inflammatory T cells following erlotinib treatment in EGFR mutant lung cancer mouse models

**Fig. 1:**
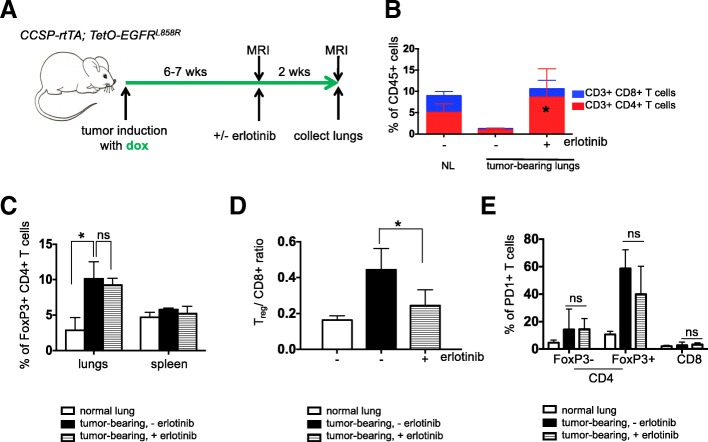
The immunosuppressive microenvironment in murine EGFR^L858R^ –induced lung adenocarcinomas is partially reversed by erlotinib. (**a**) Experimental outline of tumor induction and erlotinib treatment. *CCSP-rtTA; TetO-EGFR*^*L858R*^ mice and littermate controls on a doxycycline diet (green arrow) for 6–7 weeks were treated with erlotinib or left untreated for 2 weeks. Infiltrating immune cells were analyzed by flow cytometry. Quantification of (**b**) CD4 and CD8 T cells (**c**) FoxP3 positive CD4 T cells (**d**) T_reg_/ CD8+ T cell ratio and (**e**) PD-1 positive FoxP3- and FoxP3+ CD4 and CD8 T cells in the lungs (and spleens) of normal lung (NL) and tumor bearing *CCSP-rtTA; TetO-EGFR*^*L858R*^ mice in the absence (−) and presence (+) of erlotinib for 2 weeks. Data are obtained from three independent experiments, (*n* = 4–6 mice per group). Data are shown as mean ± SD and * is *P* < 0.05 in a student’s t-test

To evaluate the changes that occur in the immune microenvironment upon TKI treatment, *CCSP-rtTA; TetO-EGFR*^*L858R*^ bitransgenic mice on a doxycycline diet were treated with erlotinib, an EGFR TKI, for a period of 2 weeks (Fig. 1a). In six tumor bearing mice after 2 weeks of erlotinib treatment, the disease is mostly undetectable by Magnetic Resonance Imaging (MRI) (Additional file 1: Figure S1A) and largely resolved histopathologically (Additional file 1: Figure S1B). At the end of the treatment, lung and spleen single cell suspensions were prepared and analyzed by flow cytometry. We compared the immune profiles of normal healthy lungs from four mice and lungs from six tumor-bearing untreated and six erlotinib-treated mice. To ensure that the effects observed were not due to the presence of doxycycline in the mouse diet, all of the mice, including controls were maintained on doxycycline for the same amount of time. We found a consistent reduction in the fraction of CD45^+^ immune cells and the absolute number of CD4^+^ and CD8^+^ T cells per gram of lung tissue in untreated tumor-bearing lungs that was reversed upon TKI treatment (Fig. 1b and Additional file 1: Figure S1C&D).

**Fig. 2:**
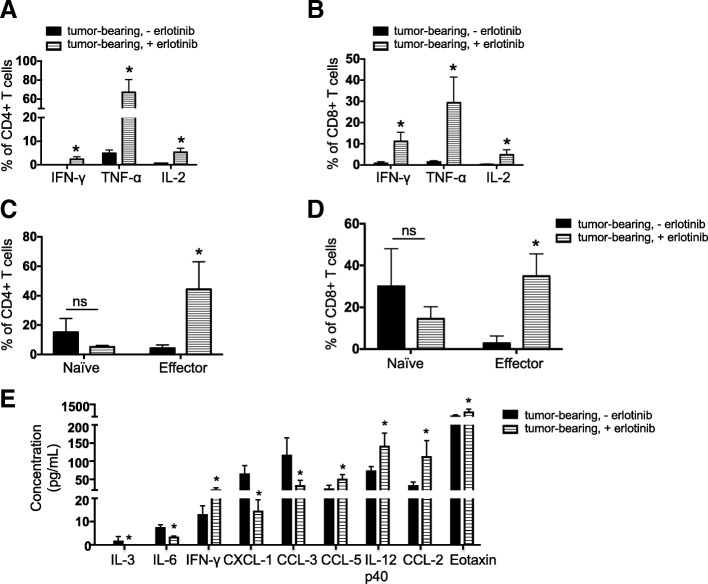
Increased production and presence of immunostimulatory cytokines following erlotinib treatment. Quantification of the levels of indicated effector cytokines from (**a**) CD4 T cells and (**b**) CD8 T cells after PMA/ionomycin stimulation and intracellular cytokine staining of cells in the lungs of tumor bearing *CCSP-rtTA; TetO-EGFR*^*L858R*^ mice in the absence (−) and presence (+) of erlotinib for 2 weeks. Quantification of naïve and effector (**c**) CD4 and (**d**) CD8 T cells in lungs of *CCSP-rtTA; TetO-EGFR*^*L858R*^ tumor bearing mice untreated or treated with erlotinib for 2 weeks. Data are from three independent experiments, (*n* = 3 mice per group) (**e**) Quantification of chemokines and cytokines in lungs of tumor bearing *CCSP-rtTA; TetO-EGFR*^*L858R*^ mice in the absence (−) and presence (+) of erlotinib for 2 weeks. Proteins (from a panel of 23) with significantly different levels between untreated and erlotinib-treated lungs are shown. Data are shown as mean ± SD and * is P < 0.05 in a student’s t-test

To determine whether there were any differences in the T cells in tumor-bearing lungs indicative of an immunosuppressive microenvironment, we quantified regulatory T cells present in the different conditions. We observed a significant increase in Foxp3^+^ regulatory T cells (T_regs_) in the lungs of tumor-bearing mice regardless of erlotinib treatment (Fig. 1c and Additional file 1: Figure S1E) suggesting that these immunosuppressive cells, which also may play a role in tissue repair, are retained even following erlotinib-mediated tumor regression. Despite the lack of a major shift in the proportion of T_regs_ in the erlotinib-treated lungs, the T_reg_/CD8^+^ T cell ratio decreased with erlotinib treatment, likely due to the increase in CD8^+^ T cells and indicative of a shift towards a more immunostimulatory microenvironment (Fig. 1d). Interestingly, these T_regs_ retained a high level of PD-1 expression that was unchanged with erlotinib treatment (Fig. 1e and Additional file 1: Figure S1F). To confirm these findings using an orthogonal approach, we used immunofluorescence to detect the tumor cell marker, cytokeratin, a pan T cell marker CD3, and the T_reg_ marker, Foxp3. We observed that erlotinib treatment induced infiltration of T cells into the lungs compared to untreated tumor-bearing lungs (Additional file 1: Figure S1G). Our quantification of Foxp3^+^ cells from these sections also revealed that there was no significant difference in their abundance between untreated and erlotinib-treated lungs (Additional file 1: Fig. S1H). In vitro T cell stimulation assays demonstrated that both CD4^+^ and CD8^+^ T cells showed increased production of the cytokines IFN-γ, TNF-α and IL-2 after erlotinib treatment indicative of an activated phenotype (Fig. 2a&b and Additional file 1: Figure S2A). These results suggest the presence of an immunosuppressive microenvironment in the lungs of mice with *EGFR*^*L858R*^ tumors, which is consistent with findings from a mouse model of *EGFR*^*Ex19del*^ mutant lung cancer [16]. Erlotinib treatment leads to an increase in the numbers of lymphocytes, their higher cytokine production and a limited reduction in the proportion of T_regs_.

To further study the properties of tumor-infiltrating T cells after erlotinib treatment, we used an in vivo labeling approach to distinguish circulating and parenchymal lung T cells from tumor-bearing mice left untreated or treated with erlotinib for 2 weeks (*n* = 3 mice per group) [31]. CD4^+^ and CD8^+^ T cells in the lungs were further classified as naïve or effector based on their expression of molecules involved in lymphocyte migration (e.g. CD62L) necessary for T cell entry into lymph nodes through high endothelial venules [32] and molecules involved in lymphocyte adhesion (e.g. CD44) required to enter sites of inflamed peripheral tissues [33], where interaction with target antigens can occur. Naïve CD4^+^ and CD8^+^ T cells, defined as CD62L^high^ CD44^low^, were unchanged after erlotinib treatment (Fig. 2c). Conversely, percentages of CD62L^low^ CD44^high^ effector CD4^+^ and CD8^+^ T cells were significantly increased after treatment (Fig. 2d), suggesting that erlotinib treatment leads to increased effector T cells in the tumor microenvironment. There was no significant difference in the expression of Granzyme B on CD4^+^ or CD8^+^ T cells from tumor bearing lungs before and after erlotinib treatment (Additional file 1: Figure S2B). Moreover, compared to a splenocyte control (Additional file 1: Figure S2C), T cells in the lungs expressed very low Granzyme B (GzmB) after in vitro stimulation. We investigated the expression of CD107a, a marker of T cell degranulation following stimulation, and observed undetectable expression. This suggests that in spite of enhanced cytokine secretion after erlotinib, the T cells in the tumor microenvironment do not degranulate. In order to further characterize lung CD4^+^ and CD8^+^ T lymphocytes, we isolated lung-resident CD4^+^ and CD8^+^ T cells and performed RNA sequencing to query their gene expression profiles. As predicted, we detected abundant expression of T cell lineage markers *Cd3e, Cd4, Cd8a* and *Cd8b* in the relevant cell populations that was unchanged by erlotinib treatment (Additional file 1: Figure S2D&E). In addition, we found that T cells from untreated tumors and erlotinib treated tumors have similar levels of expression of the T-cell co-stimulatory molecules *Cd28, Cd27* and *Icos* (Additional file 1: Figure S2D&E). Ingenuity Pathway Analysis (IPA) revealed leukocyte extravasation signaling and agranulocyte adhesion and diapedesis (extravasation) amongst the top ten pathways that changed significantly after erlotinib treatment suggesting that erlotinib treatment modulates lymphocyte properties related to movement and migration (Supplementary Table 1).

Next, to gain insight into the cytokine milieu present in EGFR mutant tumors and how this changes with erlotinib treatment, we used a multiplex immunoassay to measure the protein level of 23 cytokines from whole lung lysates of untreated and treated tumors. We found that the T cell chemoattractants CCL2 and CCL5 increased after erlotinib treatment, as did the levels of several pro-inflammatory cytokines (e.g. IFN-γ, IL-12p40) (Fig. 2e). Concomitant decreases in the cytokine CCL3 and chemokine CXCL1 were found. Overall, these data suggest that erlotinib leads to changes in the lung tumor microenvironment that are conducive to the recruitment and survival of T cells.

### Tumor regression mediated by erlotinib indirectly leads to the changes in the immune microenvironment

**Fig. 3:**
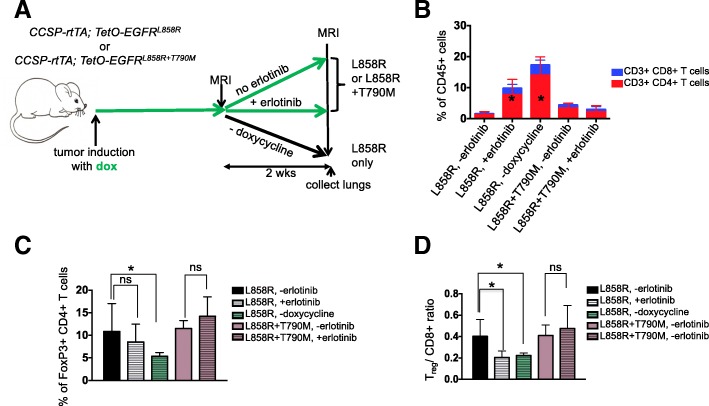
Changes in T cells in the immune microenvironment are due to tumor regression. (**a**) Experimental outline of tumor induction and erlotinib treatment. *CCSP-rtTA; TetO-EGFR*^*L858R*^ or *CCSP-rtTA; TetO-EGFR*^*L858R + T790M*^ mice and littermate controls on a doxycycline diet (green arrow) were treated with erlotinib or left untreated for 2 weeks or taken off doxycycline diet. Infiltrating immune cells were analyzed by flow cytometry. Quantification of (**b**) CD4 and CD8 T cells, (**c**) FoxP3 positive CD4 T cells and (**d**) the T_reg_/ CD8 ratio in lungs of tumor bearing *CCSP-rtTA; TetO-EGFR*^*L858R*^ or *CCSP-rtTA; TetO-EGFR*^*L858R + T790M*^ mice in the absence (−) and presence (+) of erlotinib for 2 weeks or after doxycycline withdrawal. Data are from three independent experiments, (n = 4–6 mice per group). Data are shown as mean ± SD and * is P < 0.05 in a student’s t-test

We further questioned whether the effect of erlotinib on the tumor microenvironment was a direct consequence of the TKI or an indirect result of drug-induced tumor regression. To address this question, we leveraged the inducible nature of our model system and removed doxycycline from the diet of six tumor-bearing EGFR^L858R^ mice for 2 weeks. Doxycycline withdrawal turns off the transgene initiating rapid tumor cell death similar to that observed with erlotinib (*n* = 6 mice) [26], (Fig. 3a and Additional file 1: Figs. S3A and B). As is the case with erlotinib, we saw an increase in the percentage of CD4^+^ and CD8^+^ T cells in the lungs of these models (Fig. 3b, Additional file 1: Figure S3C and D). Dox withdrawal had a more profound effect on T_regs_ which decreased significantly following oncogene de-induction (along with a corresponding decrease in the T_reg_/CD8 ratio) compared to what was observed with erlotinib treatment (Fig. 3c and d). To further explore whether tumor regression, and not erlotinib directly, was causing the observed changes in the immune microenvironment, we studied mice with *EGFR* mutant lung cancer induced by expression of the EGFR^L858R + T790M^ mutant that is unresponsive to erlotinib treatment (Additional file 1: Figs. S3A and B) [34]. Following erlotinib treatment of six L + T tumor-bearing mice we did not observe changes in the immune microenvironment (Fig. 3b, c&d). We also treated mono-transgenic (either TetO-EGFR^L858R^+;CCSP-rtTA- or TetO-EGFR^L858R^-;CCSP-rtTA+) healthy littermates with erlotinib for 2 weeks as an alternative approach to query whether the inhibitor exerts non-specific effects on immune cells and observed no differences in the immune microenvironment between erlotinib treated or untreated lungs (*n* = 4 mice per group) (Additional file 1: Fig. S3E and F). These results lead us to conclude that the changes in the immune microenvironment are not a result of a direct effect of erlotinib on immune cells but rather a consequence of the process of tumor regression itself.

**Fig. 4:**
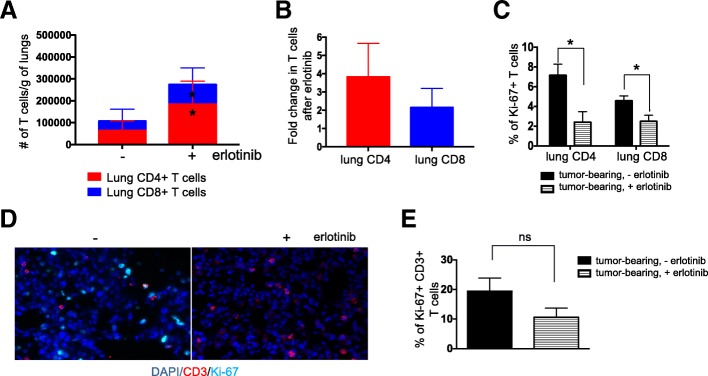
Erlotinib-mediated tumor regression increases lung T cells. (**a**) Absolute number and (**b**) Fold change in number of parenchyma lung CD4 and CD8 T cells of tumor bearing *CCSP-rtTA; TetO-EGFR*^*L858R*^ mice in the absence (−) and presence (+) of erlotinib for 2 weeks. Quantification of (**C**) Ki-67+ CD4 and CD8 T cells of tumor bearing *CCSP-rtTA; TetO-EGFR*^*L858R*^ mice in the absence (−) and presence (+) of erlotinib for 2 weeks. (**d**) Immunofluorescent (IF) stain and (**e**) quantification of CD3 T cells (red) and Ki-67 positive cells (Cyan) in lungs of tumor bearing *CCSP-rtTA; TetO-EGFR*^*L858R*^ mice in the absence (−) and presence (+) of erlotinib for 2 weeks. Nuclei were counterstained with Dapi (blue). Data are obtained from three independent experiments, (n = 4–6 mice per group). Data are shown as mean ± SD and * is P < 0.05 in a student’s t-test

To further study whether erlotinib directly affects tumor-infiltrating T cells we used in vivo labeling to distinguish circulating (i.e., cells in the vasculature) and parenchymal lung T cells followed by flow cytometry analysis. Notably, erlotinib treatment led to an increase in the absolute number of T cells present in the lung epithelium compared to untreated tumor-bearing lungs (*n* = 6 mice per group) (Fig. 4a). This translated into a 4-fold increase in CD4^+^ T cells and 2-fold increase in CD8^+^ T cells (Fig. 4b). This difference was not as prominent in the circulating T cells collected from the mouse lungs (Additional file 1: Figure S4A & B). Interestingly, the lung CD4^+^ and CD8^+^ T cells showed decreased Ki-67 positivity upon erlotinib treatment suggesting that the increased number of these cells was not due to increased proliferation following erlotinib treatment (Fig. 4c). Co-immunofluorescent staining of lung sections with antibodies against CD3 and Ki-67 showed a similar trend (Fig. 4d and e). Analogous findings were observed in samples from mice following doxycycline withdrawal (*n* = 4) supporting the possibility that the decrease in T cell proliferation is an indirect effect of the tumor regression rather than a direct effect of erlotinib on the T cells (Additional file 1: Figure S4C).

**Fig. 5:**
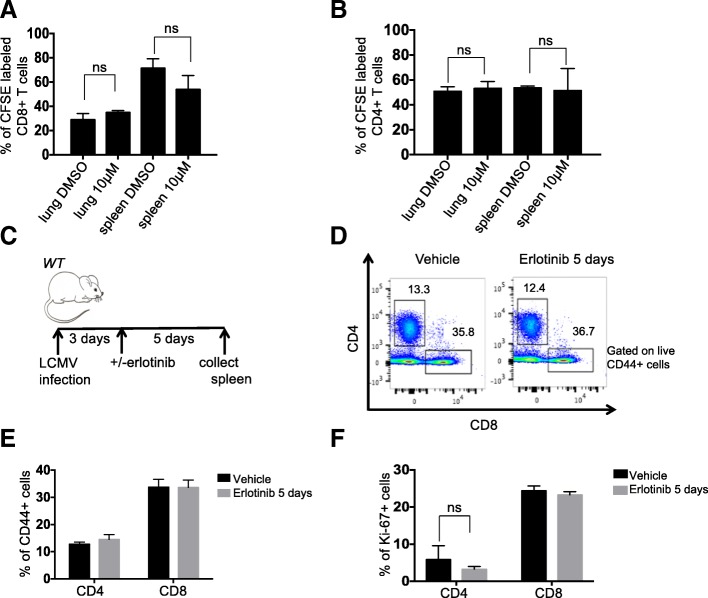
Erlotinib does not diminish T cell proliferation in vitro or in vivo. Quantification of erlotinib-treated (**a**) CD8 and (**b**) CD4 T cells isolated using magnetic beads from lungs and spleens of tumor bearing four *CCSP-rtTA; TetO-EGFR*^*L858R + T790M*^ mice and labeled with CFSE. The proportion of dividing cells was assessed 120 h after 10 μm erlotinib or DMSO treatment based on CFSE dilution. (**c**) Experimental layout of control, non-tumor bearing *CCSP-rtTA; TetO-EGFR*^*L858R*^ mice infected with LCMV for 8 days with intervening daily administration of erlotinib or vehicle for 5 days, (n = 3 mice per group). Splenic T cells were collected and analyzed by flow cytometry. (**d**) Representative FACS plot showing the percentage of CD44+ CD4+ or CD44+ CD8+ T cells and quantification of (**e**) CD44+ CD4+ or CD44+ CD8+ T cells. (**f**) Ki-67+ CD4+ or Ki-67+ CD8+ T cells from vehicle or erlotinib treated LCMV infected mice. Data are shown as mean ± SD and * is P < 0.05 in a student’s t-test

To further confirm that erlotinib did not act directly on T cells, we evaluated its effect on T cell proliferation by performing CFSE staining (Additional file 1: Figure S5A and B) of 10 μM erlotinib and DMSO-treated T cells isolated from spleens and lungs of tumor-bearing mice. We found that erlotinib, even at this high concentration, did not alter T cell proliferation in vitro (Fig. 5a, b and Additional file 1: Figure S5C). We also tested the effects of this TKI on T cells after LCMV infection in vivo (Fig. 5c) and found no effect on the abundance of CD44^+^ activated CD4^+^ or CD8^+^ T cells with erlotinib treatment (Fig. 5d&e). In addition, we did not observe a significant difference in Ki67^+^ CD4^+^ or CD8^+^ T cells between erlotinib and vehicle treated mice (*n* = 3 mice per group) suggesting that erlotinib does not affect proliferation of these cells directly (Fig. 5f).

### Erlotinib treatment leads to increased maturation of myeloid cells

**Fig. 6:**
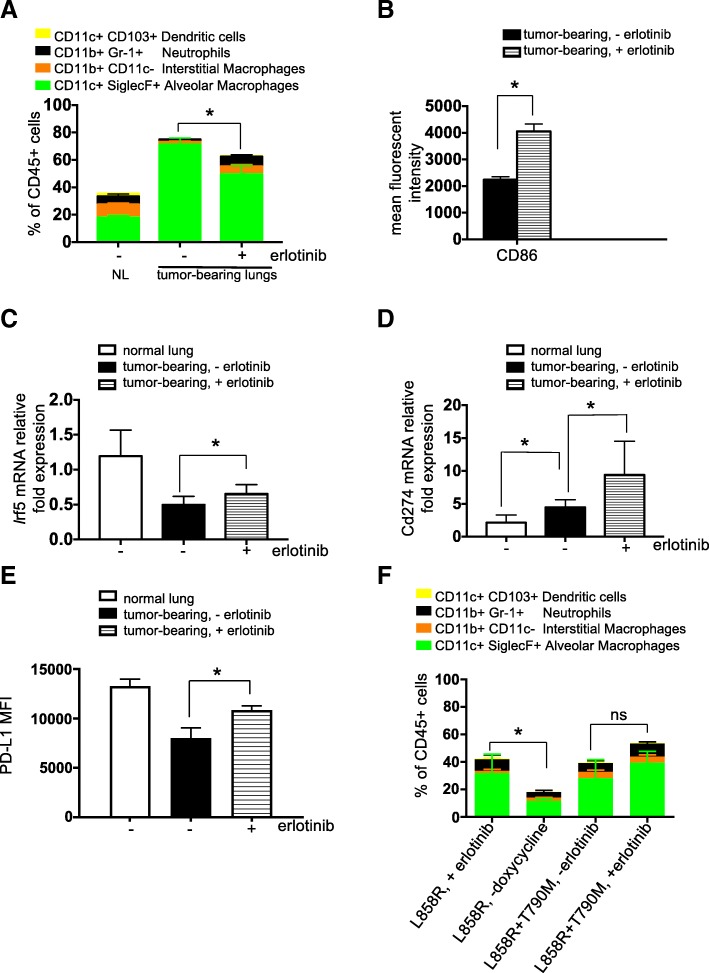
Erlotinib decreases alveolar macrophages and mediates a macrophage phenotypic switch indicative of an improved maturation. Quantification of (**a**) myeloid cell populations, (**b**) mean fluorescent intensity of the co-stimulatory molecule, CD86 in alveolar macrophages (AMs), (**c**) Irf5 and (**d**) *Cd274* mRNA expression in AMs (**E**) PD-L1 mean fluorescent intensity on AMs in lungs of control (normal) and tumor bearing *CCSP-rtTA; TetO-EGFR*^*L858R*^ mice in the absence (−) and presence (+) of erlotinib for 2 weeks. (**f**) Quantification of myeloid cell populations in lungs of tumor bearing *CCSP-rtTA; TetO-EGFR*^*L858R*^ *treated with erlotinib or* taken off doxycycline diet for 2 weeks or *CCSP-rtTA; TetO-EGFR*^*L858R + T790M*^ mice in the absence (−) and presence (+) of erlotinib for 2 weeks. Data are obtained from three independent experiments, (n = 4–6 mice per group). Data are shown as mean ± SD and * is P < 0.05 in a student’s t-test

First, we investigated the proportions of myeloid cell populations following erlotinib treatment. Specifically, we measured the percentage of alveolar and interstitial macrophages, neutrophils and dendritic cells (Fig. 6a). As observed by others [35], there was a prominent expansion of alveolar macrophages (AM) in tumor-bearing mouse lungs and this cell population was significantly decreased after erlotinib treatment (Fig. 6a) likely due to decreased proliferation of those cells as shown by a lower percentage of Ki-67^+^ positivity in that population after TKI treatment (Additional file 1: Figure S6A). In direct opposition to the pattern observed with AMs, interstitial macrophages and neutrophils were decreased in tumor-bearing lungs compared to controls and increased after erlotinib treatment, (*n* = 4–6 mice per group) (Fig. 6a). Dendritic cells were notably absent in tumor-bearing untreated lungs compared to their healthy lungs counterpart. We did observe a significant increase in CD103+ dendritic cells after erlotinib treatment (Fig. 6a).

Pulmonary AMs serve diverse roles in defense against pathogens in the respiratory tract. In addition to their well-established phagocytic roles and microbicidal functions [36], they also initiate pro-inflammatory responses through secretion of cytokines, which can stimulate T helper type 1 (T_H1_) responses or anti-inflammatory responses through secretion of IL-10 [37]. Finally, AMs have been described as poor antigen presenting cells, due to low expression of the co-stimulatory molecules CD80 and CD86 [38]. We observed an increase in the mean fluorescence intensity of CD86 on AMs suggesting a mature antigen presenting phenotype (Fig. 6b). Further supporting a switch in the macrophages to a pro-inflammatory phenotype, *Irf5* expression was increased in AMs isolated from erlotinib-treated lungs (Fig. 6c). High expression of *Irf5* has been shown to be characteristic of pro-inflammatory M1 macrophages, which are potent promoters of T_H1_ responses [39]. The levels of expression of M2 macrophage markers such as Chitinase-like 3 or MRC-1 were unchanged in lung tumors compared to healthy lungs or after erlotinib treatment. Interestingly, gene expression of *Cxcl2* increased in AMs after erlotinib treatment (Additional file 1: Figure S6B). This could potentially explain the increased neutrophils observed in TKI-treated lungs (Fig. 6a). These results suggest that erlotinib-induced tumor regression treatment triggers an inflammatory response in AMs.

Since a decrease in CD8^+^ T cell responses can be mediated by immune checkpoint ligands such as PD-Ligand 1 (PD-L1 or B7H1), we investigated whether the levels of this molecule were modulated by erlotinib. We found increased *Cd274* (the gene encoding Pd-l1) expression and Pd-l1 protein on AMs after erlotinib treatment (Fig. 6d&e), perhaps as a consequence of an adaptive immune response triggered by the inflammatory microenvironment induced by erlotinib. Moreover, IFN-γ secreted by activated effector T cells, described earlier, has been shown to induce Pd-l1 in mouse models [40]. However, we did not observe a significant difference in expression of *Cd274* on Epcam+ cells from normal lungs compared to cells from tumor bearing or erlotinib treated lungs (Additional file 1: Figure S6C). Here, we also queried whether the effect of erlotinib on myeloid cells in the tumor microenvironment was a direct consequence of TKI or an indirect result of drug-induced tumor regression. We saw decreased AMs and increased interstitial macrophages, neutrophils and dendritic cells after doxycycline withdrawal (Fig. 6f). Notably, in EGFR^L858R + T790M^ mice, there was no significant difference in any of these myeloid cell populations before and after erlotinib (*n* = 6 mice per group) (Fig. 6f), further suggesting that the changes we observed are as a result of tumor regression. In four mono-transgenic healthy littermates treated with erlotinib for 2 weeks, we observed a significant reduction in the AM population but no differences in other myeloid cell populations (Additional file 1: Figure S6D).

### Boosting T cell abundance or function does not protect erlotinib-treated mice from tumor recurrence

**Fig. 7:**
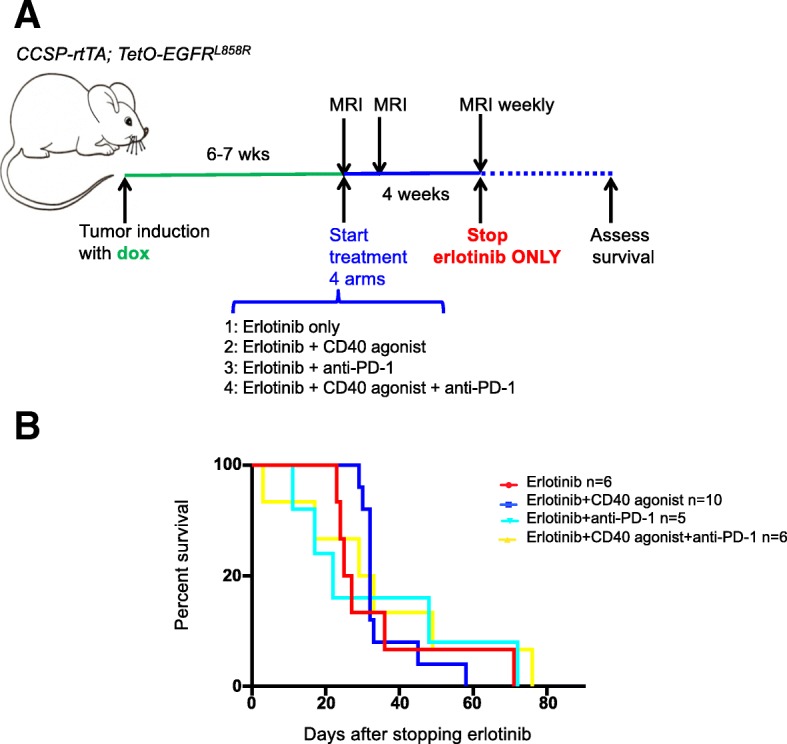
Boosting T cell function does not prevent recurrence after erlotinib treatment. (**a**) Experimental design and (**b**) survival curves of the erlotinib and immunotherapy combination study. *CCSP-rtTA; TetO-EGFR*^*L858R*^ mice were treated with erlotinib alone or in combination with immunomodulatory agents as in arms 1–4 for 4 weeks after which erlotinib was halted and immunotherapy continued until mice were moribund, (*n* = 5–10 mice per group)

Our data suggest that erlotinib largely restores the immune TME to that found in non-tumor bearing lungs, including the infiltration of cytokine-producing T cells. We wondered whether by doing this erlotinib creates the conditions for further therapeutic immune stimulation. We postulated that boosting the immune response to the tumors by targeting key molecules present on immune cells in the TME could potentially stimulate T-cell responses to the tumor cells and protect mice from tumor recurrence. To investigate this possibility, we tested the effects of therapeutic approaches to enhance T cell activity either by blocking the PD-1/PD-L1 axis using an anti-PD-1 antibody and/or using an agonistic CD40 antibody on the EGFR^L858R^-induced tumors alone or in combination with erlotinib. Agonistic CD40 antibodies have been shown to activate antigen-presenting cells, leading to a stimulation of T cell-specific antitumor responses [41] and in our models, we observed an increase in CD8^+^ T cells compared to untreated or erlotinib treated lungs (Additional file 1: Figure S7A) with the CD40 agonist, (*n* = 4–6 mice per group). Those CD8 T cells expressed higher Ki-67 and Eomesodermin (Eomes) (Additional file 1: Figure S7B&C) indicative of increased proliferation and activation of the transcriptional program necessary for the differentiation of effector CD8^+^ T cells [42]. Two-week treatment revealed that there was no difference in tumor burden between untreated tumors, anti-PD-1 and/or CD40 agonist-treated tumors (Additional file 1: Figure S7D). Not unexpectedly, given the magnitude of the effect of erlotinib on these tumors, there was not any difference in tumor regression mediated by erlotinib or erlotinib plus the anti-PD-1 and/or CD40 agonist (Additional file 1: Figure S7D&E). We then investigated whether the CD40 agonist or anti-PD-1 treatment could in combination with erlotinib delay tumor relapse. To test this, we treated tumor-bearing mice, induced with doxycycline for 6–7 weeks, with erlotinib alone or a combination of erlotinib plus the CD40 agonist or anti-PD-1 for 4 weeks (Fig. 7a), (*n* = 5–10 mice per group). As expected after 4 weeks there was no detectable tumor by MRI, with complete tumor shrinkage in all treatment groups (Additional file 1: Figure S7E). At the end of 4 weeks, the mice were taken off erlotinib but continued on the CD40 agonist, anti-PD-1 or the CD40 agonist plus anti-PD-1 (Fig. 7a). We did not see any benefit on survival or tumor burden quantified by MRI (Fig. 7b and Additional file 1: Figure S7F).

## Discussion

In this study, we investigated the changes that occur within the immune microenvironment in a mouse model of *EGFR* mutant lung cancer after treatment with the TKI erlotinib. We found that erlotinib treatment led to the re-establishment of most features of the immune microenvironment found in the lungs of healthy non-tumor bearing mice. Importantly, the erlotinib-mediated changes were not due to a direct effect of the TKIs on cells in the immune microenvironment but rather they were stimulated by the process of tumor regression itself. However, despite increases in cytokine-producing CD4 and CD8 T cells following erlotinib-treatment, combination treatment with immunotherapies like anti-PD-1 or a CD40 agonist did not effectively prevent tumor relapse.

Given the increasing interest in combining targeted therapies and immunotherapies, efforts to study the consequences of targeted therapies on the tumor immune microenvironment are growing [43]. Our findings demonstrating that erlotinib-mediated tumor regression is partially immunostimulatory are consistent with observations made with EGFR TKIs and with other targeted therapies. Studies of the BRAF inhibitor vemurafenib in a mouse model of *Braf* mutant and *Pten* deficient melanoma showed increased cytokine producing T cells in tumors following kinase inhibitor treatment [41, 44]. Similarly, activated CD8 cells were also more abundant in a *Kit* mutant gastrointestinal stromal tumor (GIST) model after imatinib treatment [45]. EGFR TKIs have also been shown to have immunostimulatory properties (that we now understand are likely due to the tumor regression that they induce). Venugopalan and colleagues demonstrated that 24 h after TKI treatment, when extensive cell death is occurring, immune cell infiltration in the lungs of mouse models of EGFR mutant lung cancer is increased [25]. Jia and others also showed an increased population of immune cells in this model after TKI treatment, with the maximum effect observed 48 h after treatment [46]. Prior to our study, the consequences of TKIs like erlotinib on the immune microenvironment after maximum tumor regression had not been examined. Since TKIs are administered daily and patients receive these therapies continuously, understanding the longer-term consequences of these drugs on the immune microenvironment is critical. The immune cell infiltration patterns found at 24 h [25] and 2 weeks (in our study) are similar consistent with the possibility that the process of tumor regression serves as a trigger for these changes. These indications of immune activation were counterbalanced by data indicating that after erlotinib treatment the tumors retained some immunosuppressive properties including abundant regulatory T cells (Fig. 1c) and increased levels of PD-L1 (Fig. 6d and e). While the T_regs_ may be indicative of immunosuppression persistent after erlotinib, the cells may also be playing a role in tissue repair after inflammation [47]. Whether targeting these elements of immunosuppression would be an effective strategy to slow tumor growth is currently unknown and actively being investigated. Such studies could include direct targeting of T_regs_ either by using antibodies such as ipilimumab (anti-CTLA-4) that can deplete T_regs_ [48] or, in genetically engineered mouse models, by ablating T_regs_ [49]. PD-1 axis inhibitors have been shown to modestly prolong survival of mice with *EGFR* mutant lung cancer [16], however, whether in combination with erlotinib this translates into improved survival and/or delays the emergence of resistance is unknown. In patients, the response rate of *EGFR* mutant tumors to PD-1 or PD-L1 blockade is below 10% and therefore lower than in NSCLC as a whole (RR ~ 20%) potentially due to the lower immunogenicity of the tumors mainly arising in former/never smokers and having a low mutational background [14, 50–52]. Large studies of TKIs in combination with checkpoint inhibitors have not been conducted to date in part due to concerns regarding the toxicity of these combinations. However, in a small study of erlotinib in combination with nivolumab, the combination was well-tolerated and the response rate to the combination in the refractory setting was 15% suggesting that some patients benefit from these agents [17]. We attempted to determine whether leveraging the immune cell changes in the TME mediated by erlotinib with an immunotherapeutic agent like anti-PD-1 or an agonistic CD40 antibody could further stimulate the immune system to exert anti-tumor effects. We found that addition of these agents to erlotinib treatment did not prevent or delay tumor relapse. These data indicate that the tumors are refractory to T-cell mediated killing even when T cells are abundant and not exhausted. It has been established that lung tumors in genetically engineered mouse models, including the EGFR^L858R^ model we used, have a significantly lower frequency of nonsynonymous mutations compared to human lung adenocarcinomas [53, 54]. The low frequency of somatic mutations that arise during tumor development in these models lead to the generation of few neoantigens to induce T cell responses. This may explain the lack of a strong T cell-mediated immune response in this tumor model [55]. Future studies aimed to study antigen-specific T cell responses in new systems that express model antigens and/or have higher mutation burdens more reflective of human lung cancer are ongoing. An alternative but not mutually exclusive possibility is that multiple immunosuppressive pathways active in the tumors need to be simultaneously inhibited to engage the immune system. This is supported by our data showing that T_regs_ represent a significant fraction of T cells that are present in EGFR^L858R^-induced tumors following erlotinib treatment. The extent to which these signals play a role in tumorigenesis and need to be reversed for tumor regression is still poorly understood.

There are several ways in which targeted therapies may be affecting immune cells. They could either be acting directly via on-target or off-target activities on immune cells present in the tumor. Alternatively, the changes could be an indirect consequence of the biological effects (e.g induction of apoptosis) of targeted therapies. Indeed, forms of cell death, like necrosis, have long been recognized as having potentially immunogenic consequences, and data suggest that apoptosis could also have immunological effects [56]. In support of this, our study provides evidence that the TKI erlotinib itself does not act directly on immune cells in the tumor microenvironment but rather changes in immune infiltrates result indirectly from the process of tumor regression. First, we found that in a mouse model of erlotinib-resistant lung cancer in which tumors do not regress upon treatment with the TKI, low numbers and functionally impaired CD4 and CD8 lymphocytes are found similar to untreated tumors even following TKI treatment. Second, erlotinib did not affect the proportion of lymphocytes in the lungs of healthy non-tumor bearing mice. Third, erlotinib treatment of lymphocytes isolated from tumor-bearing mouse lungs or from spleen does not affect their proliferation or activation. Others have shown that erlotinib does inhibit the proliferation of T cells isolated from mouse lymph nodes [23]. It is possible that these differences are due to the different biological contexts examined, namely lung or spleen cells from tumor-bearing or LCMV-infected mice as opposed to T cells from wild-type lymph nodes. Erlotinib has also been shown to act directly on tumor cells by increasing MHC I antigen presentation rendering them more responsive to T-cell mediated attack [57]. However, it is unclear whether such mechanisms would be in play in *EGFR* mutant tumor cells that are undergoing apoptosis but rather in *EGFR* wild-type tumor cells where erlotinib does not lead to cell death.

Our study has several translational implications. First, the data underscore the difficulty of harnessing CD8 T cell cytotoxicity in the context of poorly antigenic tumors like those present in these mouse models of EGFR mutant lung cancer. It is possible that strategies to leverage the immune system that do not rely on CD8 T cells may be more successful in these tumors such as targeting innate immune cells. Indeed, depletion of alveolar macrophages has been shown to reduce tumor burden in these models [35] suggesting that targeting these cells may be an avenue for therapeutic benefit. Second, our study highlights how the process of tumor regression itself leads to the observed changes in the tumor immune microenvironment rather than representing a direct effect of erlotinib on immune cells. Understanding the contributions of individual drugs to the tumor immune microenvironment can be important for selecting therapeutic combinations to maximize efficacy and minimize toxicity. In the case of EGFR mutant lung cancer, where there are concerns about combining TKIs with immunotherapies, like immune checkpoint inhibitors due to toxicity, it is possible that other agents that lead to tumor regression could be used. This could be relevant in tumors resistant to TKIs when TKI treatment is no longer an option and other approaches need to be explored.

A limitation of our study is the absence of confirmatory evidence of our findings in TKI-responsive tumor specimens from patients. Such samples are challenging to obtain because biopsies are not routinely performed when a tumor is responding to therapy. Future clinical trials of TKIs that include on-treatment biopsies like the ELIOS Study (NCT03239340) will be valuable to evaluate TKI-induced changes in the tumor microenvironment in human tumors. An additional limitation of our study is the low mutation burden of tumors in genetically engineered mouse models [53]. Even though our model provides a physiologically relevant tumor microenvironment, the low frequency of somatic mutations that arise during tumor development limits the number of neoantigens that can induce T cell responses.

## Conclusions

Altogether, our findings lay the foundation for understanding how TKIs modulate the tumor immune microenvironment and their association with the process of tumor regression. These studies also provide us with insight into the features of the immune tumor microenvironment under continuous TKI exposure and whether these can be leveraged therapeutically.

## Additional file

Additional file 1: **Figure S1**. MRI images, histology and representative flow cytometry plots of normal or tumor-bearing lungs before and after erlotinib. (**A**) Coronal images of *CCSPrtTA; TetO-EGFR*^L858R^ mouse lungs before (left panel) and after (right panel) treatment with erlotinib. (**B**) Hematoxylin and eosin (H&E) stain of lungs from control (normal) and tumor bearing *CCSP-rtTA; TetO-EGFR*^L858R^ mice in the absence (−) and presence (+) of erlotinib for 2 weeks. Bar: 50 μm. Absolute number of (**C**) CD4 and (D) CD8 T cells normalized to weight of lungs of control (normal) and tumor bearing *CCSP-rtTA; TetO-EGFR*^L858R^ mice in the absence (−) and presence (+) of erlotinib for 2 weeks. Representative FACS plot showing percentage of (**E**) FoxP3+ and FoxP3- CD4+ T cells (**F**) PD1+ FoxP3+ T cells. (**G**) Immunofluorescence (IF) stain of lung epithelial cells (green), CD3 T cells (red) and FoxP3 Tregs (Cyan). Nuclei were counterstained with Dapi (blue) (**H**) Quantification of FoxP3+ CD3 T cells in lung tumor bearing *CCSP-rtTA; TetO-EGFR*^L858R^ mice in the absence (−) and presence (+) of erlotinib for 2 weeks stained by IF. Data are shown as mean ± SD and * is *P* < 0.05 in a student’s t-test. NS, non-significant. **Figure S2**. Representative flow cytometry plots of cytokine producing T cells and gene expression profile of T cells isolated from tumor-bearing lungs before and after erlotinib. Representative FACS plots showing the percentage of (**A**) TNF-α+, IFN-γ+, and IL-2+ CD4 T cells. (**B**) Quantification of GzmB+ CD4 and CD8 T cells after PMA/ionomycin stimulation and intracellular cytokine staining of cells in the lungs of tumor bearing *CCSP-rtTA; TetO-EGFR*^L858R^ mice in the absence (−) and presence (+) of erlotinib for 2 weeks. (**C**) Representative FACS plot showing percentage of GzmB+ splenic CD8 T cells after PMA/ionomycin stimulation and intracellular cytokine staining of cells. (**D**) CD8 and (**E**) CD4 T cells isolated from *CCSP-rtTA; TetO-EGFR*^L858R^ tumor bearing mice untreated or treated with erlotinib. Heatmap was generated using normalized expression values. NS, non-significant. **Figure S3**. MRI images, histology and representative flow cytometry plots of erlotinib sensitive and resistant tumors. (**A**) Coronal images of: *CCSP-rtTA; TetO-EGFR*^L858R^ mouse lungs before (left panel) and after (right panel) cessation of doxycyline and *CCSP-rtTA; TetO-EGFR*^L858R + T790M^ mouse lungs before (left panel) and after (right panel) treatment with 2 erlotinib. (**B**) Hematoxylin and eosin (H&E) stain of lungs from tumor bearing: *CCSP-rtTA; TetO-EGFR*^L858R^ untreated or taken off doxycycline diet for 2 weeks and *CCSP-rtTA; TetOEGFR*^L858R+ T70M^ mice in the absence (−) and presence (+) of erlotinib for 2 weeks. Bar: 50 μm. Absolute number of (**C**) CD4 and (**D**) CD8 T cells normalized to weight of lungs of tumor bearing *CCSP-rtTA; TetO-EGFR*^L858R^ or *CCSP-rtTA; TetO-EGFR*^L858R + T790M^ mice in the absence (−) and presence (+) of erlotinib for 2 weeks or taken off doxycycline diet. Data are obtained from three independent experiments, (*n* = 4–6 mice per group) * is *P* < 0.05 in a student’s t-test. Quantification of (**E**) CD4 and CD8 T cells and (**F**) FoxP3 positive CD4 T cells in the lungs of control (normal) mice in the absence (−) and presence (+) of erlotinib for 2 weeks. Data are shown as the mean ± SD. NS, non significant. **Figure S4**. Quantification of circulating and proliferating T cells. (**A**) Absolute number and (**B**) Fold change in number of circulating lung CD4 and CD8 T cells of tumor bearing *CCSP-rtTA; TetO-EGFR*^L858R^ mice in the absence (−) and presence (+) of erlotinib for 2 weeks. (**C)** Ki-67+ CD4 and CD8 T cells of tumor bearing *CCSP-rtTA; TetO-EGFR*^L858R^ mice in the absence (−) and presence (+) of erlotinib for 2 weeks or mice taken off doxycycline for 2 weeks. Data are shown as the mean ± SD and * is P < 0.05 in a student’s t-test. NS, non-significant. **Figure S5**. Experimental outline of CFSE labeling and analysis. (**A**) Flow chart for isolation, labeling and treatment of T cells from lungs and spleens of tumor bearing *CCSP-rtTA; TetO-EGFR*^L858R^ mice*.* FACS plot showing (**B**) as a control for the technique, unlabeled vs CFSE labeled splenocytes (Day 0) and (**C**) Untreated CD4 and CD8 T cells from lungs and spleens at Day 0 as well as CD4 and CD8 T cells from lungs and spleens treated with 10 μm erlotinib or DMSO after 5 days (120 h). **Figure S6**. Quantification of proliferating alveolar macrophages and myeloid cells in healthy lungs before and after erlotinib treatment. (**A**) Ki-67 positive AMs, (**B**) *Cxcl2 *expression in AMs, (**C**) *Cd274* mRNA expression in Epcam+ tumor cells from lungs of control (normal) and tumor bearing *CCSP-rtTA; TetO-EGFR*^L858R^ mice in the absence (−) and presence (+) of erlotinib for 2 weeks. (**D**) Quantification of myeloid cell populations in the lungs of control (normal) mice in the absence (−) and presence (+) of erlotinib for 2 weeks. Data are shown as the mean ± SD and * is P < 0.05 in a student’s t-test. NS, non-significant. **Figure S7**. Tumor volume measurements and survival analysis. Quantification of (**A**) CD8+ T cells, (**B**) Ki-67+ CD8+ T cells and (**C**) Eomes+ CD8+ T cells in the lungs of tumor bearing *CCSP-rtTA; TetO-EGFR*^L858R^ mice in the absence (−) and presence (+) of erlotinib, CD40 agonist or erlotinib plus the CD40 agonist for 2 weeks, (n = 4–6 mice per group). (**D**) Tumor volume quantified at 1 and 2 weeks after treatment measured by MRI normalized to pretreatment tumor volume. Change in tumor volume (**E**) pre-treatment and 1–4 weeks after treatment with erlotinib alone or erlotinib and immunotherapy combination and (**F**) pretreatment, 1–4 weeks after treatment with erlotinib alone or erlotinib and immunotherapy combination and 1–3 weeks after stopping erlotinib (relapse), (*n* = 5–10 mice per group). Data are shown as the mean ± SD and * is *P* < 0.05 in a student’s t-test. NS, non-significant. (XLSX 66 kb)

## Data Availability

The datasets used and/or analysed during the current study are available from the corresponding author opon request.

## Abbreviations

AM: Alveolar Macrophage
EGFR: Epidermal Growth Factor Receptor
Gzmb: Granzyme B
MRI: Magnetic Resonance Imaging
T_H1_: T helper type 1
TKI: Tyrosine kinase inhibitor
TME: Tumor microenvironment
T_reg_: Regulatory T cell
